# Surgical Outcome of Vitreomacular Traction Associated With Macular Hole

**DOI:** 10.7759/cureus.32620

**Published:** 2022-12-17

**Authors:** Mashal Tayyab, Kashif Iqbal, Muhammad Awaid Abid, Fawad Ur Rahman, Hamza A Tayyab

**Affiliations:** 1 Ophthalmology, Layton Rahmatullah Benevolent Trust Eye Hospital, Lahore, PAK

**Keywords:** internal limiting membrane peel, pars plana vitrectomy, optical coherence tomography, macular hole, vitreomacular traction syndrome

## Abstract

Vitreomacular Interface pathology and its surgical treatment is an ever-evolving field in vitreoretinal surgery. Various treatments have been proposed for macular holes associated with vitreomacular traction including ocriplasmin injection, gas injection, and pars plana vitrectomy with posterior hyaloid face stripping with or without internal limiting membrane peeling. The time of intervention in patients with vitreomacular traction syndrome is also a point of contention among researchers. Here we present a case of an 83-year-old male patient who presented to the outpatient department with a history of grossly decreased visual acuity of counting fingers in the right eye. An altered foveal reflex was seen in an otherwise unremarkable ocular examination. The left eye revealed no pertinent findings. The patient was diagnosed with vitreomacular traction syndrome on spectral domain optical coherence tomography. There was an associated grade 1b macular hole according to the International Vitreomacular Traction Study classification. As the roof of the macular hole was intact, we decided to proceed with pars plana vitrectomy and careful stripping of the posterior hyaloid face. However, this resulted in a full-thickness macular hole and no change in visual acuity. A second surgery comprising internal limiting membrane peel using brilliant blue dye with perfluoropropane (C_3_F_8_) gas tamponade was done. Follow-up after six weeks showed a visual acuity improvement to 20/120 and restoration of foveal configuration. To the best of our knowledge, such a clinical case has not been reported in locally published literature.

## Introduction

Vitreomacular traction syndrome (VMTS) is defined as an incomplete posterior vitreous detachment (PVD) with an abnormally strong adherence to the macula. This results in morphologic and functional defects due to anteroposterior traction forces [1].

The area of vitreous traction can be focal (< 1500 micrometres) or broad (>1500 micrometres). Focal vitreous traction increases on saccades. This can result in foveal cysts, which can progress to an idiopathic macular hole or foveal detachment. The resulting metamorphopsia and decreased visual acuity give rise to the need for intervention. Many treatments have been proposed for visual disturbance due to VMTS. Research shows that initial observation was generally favourable in asymptomatic patients or those with minimal symptoms of vitreomacular traction (VMT) [2].

Ocriplasmin has been demonstrated as an effective minimally invasive therapy for VMT release and full-thickness macular hole (FTMH) closure, with patients on average experiencing significant improvement in visual acuity [3]. Pneumatic vitreolysis is also a viable option for treating VMT [4].

Another option is minimally invasive pars plana vitrectomy (PPV) with posterior hyaloid face stripping to release macular traction in symptomatic VMT patients with or without macular holes. Vitreoretinal surgery effectively removes traction and gives a high closure rate of an FTMH (90-100%). It is now a very safe procedure with few side effects [5]. As published in previous studies, minimally invasive PPV was deemed to result in spontaneous restoration of the foveal architecture with improvement in best-corrected visual acuity (BCVA) [6-7].

However, posterior hyaloid face stripping in this patient resulted in FTMH with everted margins and no improvement in visual acuity. Thus, we resorted to the conventional procedure for FTMH, that is, internal limiting membrane (ILM) peel using brilliant blue dye with perfluoropropane (C_3_F_8_) gas tamponade. Subsequent optical coherence tomography (OCT) showed a restored foveal contour. The patient also showed clinical improvement in visual acuity.

## Case presentation

An 83-year-old male patient presented with a gradual, progressive decrease of vision in the right eye over three months. There was accompanying metamorphopsia. It did not vary with the time of day and was not associated with flashes, floaters or any other symptoms. Past ophthalmic history was positive for bilateral cataract extraction with non-complicated phacoemulsification and foldable posterior chamber intraocular lenses. There was no history of trauma, glasses use, or refractive surgery. The patient had no known comorbidities. Family history, social history, and drug history were also unremarkable.

A comprehensive ophthalmic examination revealed an unaided visual acuity of counting fingers (CF) in the right eye (OD) and 20/60 in the left eye (OS). BCVA remained OD CF and OS 20/40 with a manifest refraction of -1.00 *140 D. Intraocular pressure was OD 14mmHg and OS 16mmHg.

**Figure 1:**
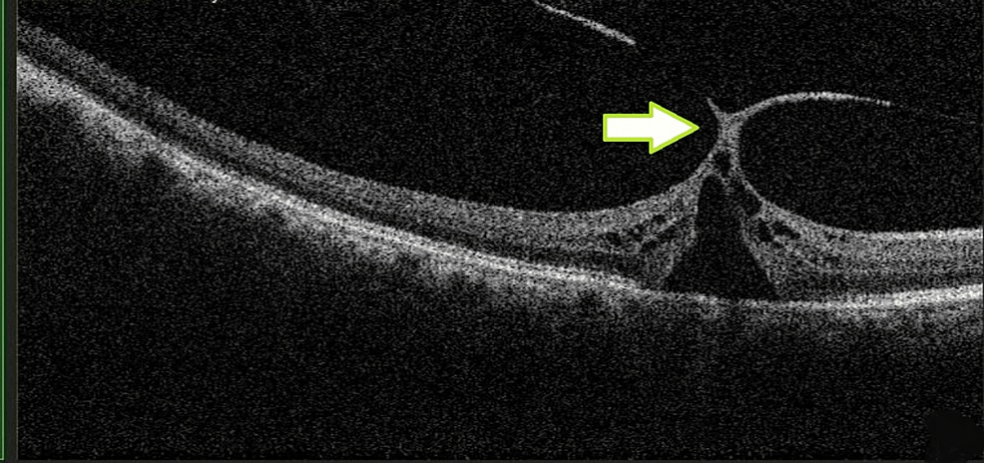
Optical coherence tomography showing vitreomacular traction (green arrow) with separation of a portion of the neurosensory retina from the retinal pigment epithelium (grade 1b macular hole on IVTS classification). IVTS: International Vitreomacular Traction Study

The patient was orthophoric with no abnormality of extraocular movements. Pupils were round and regular, and the relative afferent pupillary defect was negative. Slit lamp examination of the anterior segment was unremarkable in both eyes with no evidence of inflammation. Both eyes had well-centred posterior chamber intraocular lenses. The posterior capsule was intact bilaterally. Fundus examination of the right eye revealed a cup/disc ratio (CDR) of 0.3 with an altered foveal reflex. Retinal vasculature and peripheries were normal. The left fundus had a cup/disc ratio of 0.3 with an unremarkable fovea, vessels, and periphery. Spectral-domain optical coherence tomography (SD-OCT) (RS-3000 Advance, Nidek Co., Ltd, Gamagori, Aichi, Japan) showed grade 1b macular hole with focal VMT (Figure 1) in the right eye.

**Figure 2:**
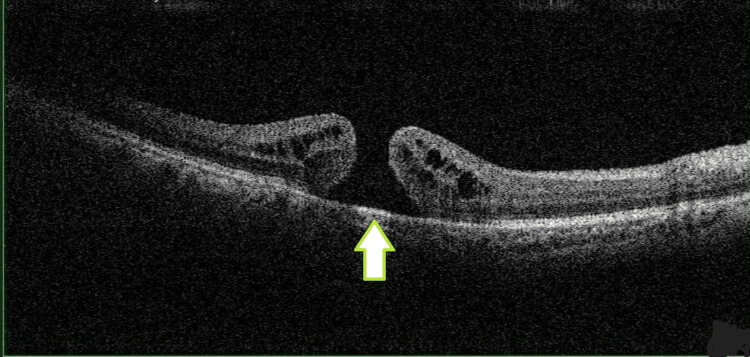
A full-thickness macular hole (green arrow) with everted margins and small cystic spaces in the neurosensory retina surrounding the defect.

Left eye OCT was unremarkable. PPV with posterior hyaloid face stripping to induce posterior vitreous detachment and air tamponade was done. Post-operative OCT done after 10 days showed development of an FTMH, 422 micron in size with everted edges (Figure 2).

**Figure 3:**
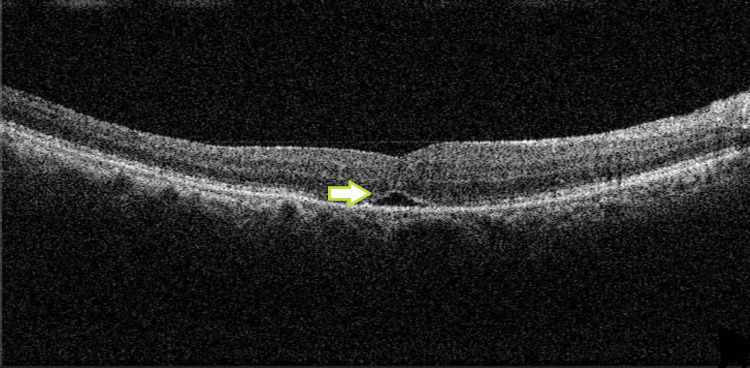
Restoration of foveal contour with a small subfoveal pigment epithelial detachment (green arrow).

VA also remained CF. We proceeded to a second surgery, which included a PPV and ILM peel with brilliant peel dye and 14% C_3_F_8 _gas tamponade. Postoperative assessment at six weeks showed visual acuity of OD 20/120 plano on BCVA and anatomical restoration of foveal configuration (Figure 3).

## Discussion

The objective of VMT treatment is to relieve the retinal traction caused by the abnormally adherent vitreous. In a small number of cases, VMT resolves spontaneously without intervention [8]. However, a study shows that 64% of eyes with VMT had a further deterioration in vision >2 lines without vitrectomy intervention [9].

As in our case, poor visual acuity, metamorphopsia, and worsening quality of life necessitated surgical intervention. Research has shown that stripping of the posterior hyaloid face to relieve VMT resulted in an improvement of visual acuity from 20/63 to 20/36 three months after the operation [10].

Other studies show visual and anatomical improvements based on the preoperative anatomical configuration. Improvement in vision was significantly greater in the eyes in focal VMT (2.82 +/- 1.47 lines) than in those with broad VMT (0.83 +/- 1.17 lines) or broad VMT with epiretinal membrane (1.29 +/- 0.49 lines) [11]. Another study shows that cases with focal VMT had greater visual improvement (P = 0.027) because the preoperative BCVA was significantly lower in the focal group (P =0.007) [12].

A similar case report of a patient with VMT associated with lamellar hole shows spontaneous closure after surgical intervention with posterior vitreous detachment with SF_6_ tamponade. In this patient, visual acuity improved from 20/60 to 20/30 with the restoration of anatomic configuration [13].

As our patient had focal VMT with stage 1b macular hole, we were anticipating good visual and anatomic outcomes following surgical intervention. We proceeded to do 23 gauge PPV with posterior hyaloid face stripping and air tamponade. A postoperative surprise was discovered on OCT done 10 days after PPV, in the form of an FTMH, 422 micron in size, with everted margins but no VMT. Another case report showed a similar complication of a patient who had undergone the same surgical intervention. However, this patient had a preoperative lamellar hole with an open roof and VMT [14].

As the visual acuity in our patient also showed no improvement from CF, we proceeded to do a second procedure encompassing PPV with ILM peel using brilliant blue dye and 14% C_3_F_8_ gas tamponade. visual acuity was checked six weeks after this procedure followed by an OCT. Visual acuity improved to 20/120 and OCT also showed restoration of the foveal contour with a small pigment epithelial detachment at the fovea.

The closure rate of macular holes after ILM peel is 95.1% [15], as was further proved by our case. However, in the interest of avoiding the surgical complications of working in close proximity to the retina as in membrane peeling and the success rates of minimally invasive PPV in VMT-associated foveal pathology, the latter approach is preferred by the authors.

## Conclusions

Our case suggests that surgical intervention for VMT yields good anatomic and visual outcomes. We conclude that even though careful posterior hyaloid face stripping in a VMT patient is a recommended intervention, if a full-thickness macular hole albeit small is observed during surgery, ILM peel should be undertaken in the first procedure instead of waiting for spontaneous closure of the defect.
